# Bacterial Meningitis and *Haemophilus influenzae* Type b Conjugate Vaccine, Malawi

**DOI:** 10.3201/eid1704.101045

**Published:** 2011-04

**Authors:** David W. McCormick, Elizabeth M. Molyneux

**Affiliations:** Author affiliations: University of Michigan, Ann Arbor, Michigan, USA (D.W. McCormick);; College of Medicine, Blantyre, Malawi (E.M. Molyneux)

**Keywords:** Bacterial meningitis, bacteria, Haemophilus influenzae type b, Hib, vaccines, epidemiology, Malawi, HIV, dispatch

## Abstract

A retrospective database review showed that *Haemophilus influenzae* type b conjugate vaccine decreased the annual number of cases of *H*. *influenzae* type b meningitis in children in Blantyre, Malawi. Among young bacterial meningitis patients, HIV prevalence was high (36.7% during 1997–2009), and pneumococcus was the most common etiologic agent (57% in 2009).

Acute bacterial meningitis (ABM) is a major cause of illness and death in children in sub-Saharan Africa (*1**,**2*). *Neisseria meningitidis* is the most common cause of ABM in the meningitis belt (sub-Saharan Africa), and *Streptococcus pneumoniae* and *Haemophilus influenzae* type b (Hib) are the most common causes in southern and eastern Africa (*1**–**4*). Of 114 case-patients with meningitis and positive cerebrospinal fluid (CSF) cultures who came to the Queen Elizabeth Central Hospital (QECH) in Blantyre, Malawi (southeastern Africa), during 1996–1997, more than half of these cases were caused by *S. pneumoniae*, Hib, or *Salmonella* spp (*5*).

In February 2002, Malawi introduced Hib conjugate vaccine in a pentavalent formulation that includes vaccine against diphtheria, pertussis, tetanus, and hepatitis B. There was no mass campaign or catch-up program. This vaccine is given routinely to patients at 6, 10, and 14 weeks of age; vaccination coverage has been ≈90% since 2002 (http://apps.who.int/immunization_monitoring/en/globalsummary/countryprofileresult.cfm). Incidence of Hib meningitis decreased but the long-term effect of the vaccination program remains unclear (*6*). We examined the effectiveness of Hib conjugate vaccine by conducting a retrospective database review of children with ABM who came to QECH in Blantyre, Malawi during 1997–2009.

## The Study

QECH is the district hospital for Blantyre District and a major referral center serving southern Malawi. We aggregated data from 3 studies of childhood ABM at QECH, where patients 2 months to 15 years of age with suspected ABM were treated (*7*; E.M. Molyneux, unpub. data). A patient with ABM was defined as a person whose CSF sample at the time of hospital admission contained >100 leukocytes/high-power microscopic field or demonstrable organisms by Gram stain or culture.

Data collection began in July 1997 and continued through December 2009. We multiplied the observed number of cases in 1997 by 2 to estimate the total number of cases for 1997. No data were collected during October 2000–October 2001; data from 2001 were excluded from analysis. Total number of cases in 2000 was estimated by multiplying the observed number of cases in 2000 by 1.25. *S. pneumoniae* and Hib had mild seasonal variations that did not affect the results, and we conclude that this estimation enables valid comparisons. We did not ascertain vaccination status of study participants.

Data were analyzed by using STATA 10.1 SE (Stata Corp LP, College Station, TX, USA). A 2-tailed t-test was used to compare mean case counts, and 2-tailed *z* tests were used to compare proportions. All studies were reviewed and approved by the College of Medicine Research and Ethics Committee in Blantyre, Malawi.

CSF samples were obtained aseptically before administration of antimicrobial drugs, labeled, and immediately sent to the laboratory. These specimens were cultured onto blood and chocolate agar plates and incubated at 37°C for 72 hours. Isolates were identified by using standard procedures (*8*). Commercial slide agglutination tests were used to serotype *H. influenzae* isolates (MAST Diagnostics, Bootle, UK). If CSF specimens were culture negative and gram negative after 2 days, they were tested for 5 common bacterial antigens (Hib, *S. pneumoniae*, *N. meningitidis*, group B streptococci, and *Escherichia coli*) by using latex agglutination reagents (Murex, Kent, UK) according to the manufacturer’s instructions.

Serum samples were tested for HIV by using >2 of the following tests: Serodia-HIV particle agglutination (Fujirebio Inc., Tokyo, Japan and Mast Diagnostics), HIVSPOT (Genelabs Diagnostics, Singapore), Determine-HIV (Abbott Laboratories, Abbott Park, IL, USA), and Capillus-HIV (Cambridge Diagnostics, Galway, Ireland). Discordant test results were confirmed by using a third test or PCR for HIV. Children <15 months of age with positive antibody test results had their serostatus confirmed by PCR.

There were 1,740 children with bacterial meningitis at QECH during 1997–2009. Their ages ranged from 2 months to 15 years (median 18 months, mean 42 months). One fourth of the children were <6 months of age and 53.8% were boys.

HIV serostatus was available for 1,486 patients; of these patients, 36.7% were HIV seropositive. This proportion increased from 30.4% (207/680) during 1997–2002 to 42% (336/801) during 2003–2009 (p<0.0001). The proportion of seropositive patients was similar for both sexes (girls, 271/696 [38.9%]; boys, 270/781 [34.6%]; p = 0.08). HIV test results were equivocal for 1 patient who was excluded from further analyses of HIV serostatus. On the basis of projections, ≈111,510 children <15 years of age were HIV seropositive in Malawi in 2009 (*9*). Census data show that there were 6,749,800 children <15 years of age in Malawi in 2009 (*10*). We estimate that 1.65% of children in Malawi are HIV seropositive. The proportion of HIV-seropositive children was significantly higher in our study population than in the general population (p<0.0001).

The number of annual cases for each causative agent of ABM changed dramatically during 1997–2009 (Figure). Before Hib vaccine was available (1997–2002), Hib was responsible for 53.2 annual cases of bacterial meningitis. After introduction of the vaccine, mean number of annual cases decreased to 9.7 (p<0.0001; Table). Mean age of these patients increased from 14 months (range 2–96 months, median 9 months) during 1997–2002 to 32 months (range 2–120 months, median 21 months) during 2003–2009 (p<0.0001).

**Figure:**
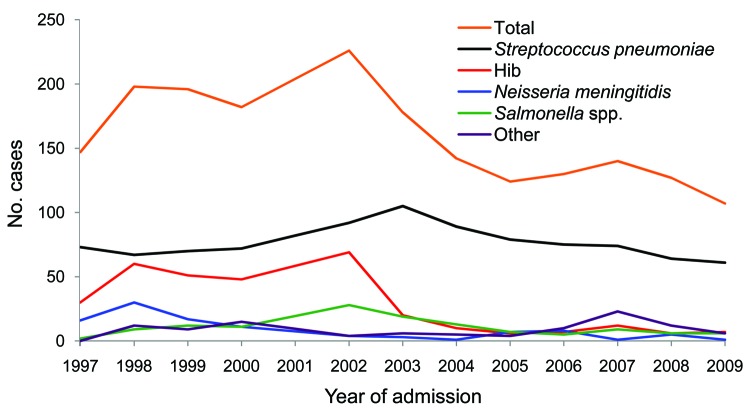
Annual number of cases of culture-positive bacterial meningitis in children, Queen Elizabeth Central Hospital, Blantyre, Malawi, 1997–2009. Data from 2001 are excluded. Hib, *Haemophilus influenzae* type b; Other, *Klebsiella* spp., *Staphylococcus aureus*, *Staphylococcus epidermidis*, *Escherichia coli*, *Brevundimonas vesicularis*, *Pseudomonas aeruginosa*, *Streptococcus pyogenes*, *H. influenzae* type C, *H. influenzae* not typed, group B streptococci, group A streptococci*,* and other species.

**Table:** Causes of bacterial meningitis among patients at Queen Elizabeth Central Hospital, Blantyre, Malawi 1997–2009

Culture organism	No. cases (annual mean ± SE)		p value
	1997–2002*	2003–2009	
*Streptococcus pneumoniae*	373 (74.6 ± 4.83)	547 (78.1 ± 5.69)	0.66
*Haemophilus influenzae* type b	266 (53.2 ± 5.51)	68 (9.7 ± 1.91)	<0.0001
*Neisseria meningitides*	78 (15.6 ± 4.27)	26 (3.7 ± 1.12)	0.0106
*Salmonella* spp.†	62 (12.4 ± 4.27)	65 (9.3 ± 1.91)	0.48
Other‡	40 (8.0 ± 2.70)	66 (9.4 ± 2.51)	0.71
No growth	100 (25.8 ± 3.40)	205 (25.1 ± 1.65)	0.85

*Excludes data from 2001. †*Salmonella enterica* serovar Typhimurium (79), *S. enterica* serovar Enteriditis (22), *S. enterica* serovar Typhi (14), and other *Salmonella* spp. (9). These numbers reflect actual case counts. Numbers were adjusted for missing data. ‡Includes *Klebsiella* spp. (4), *Staphylococcus aureus* (5), *Staphylococcus epidermidis* (2), *Escherichia coli* (5), *Brevundimonas vesicularis* (1), *Pseudomonas aeruginosa* (2), *Streptococcus pyogenes* (6), *H. influenzae* type C (4), *H*. *influenzae* nontype b (6), group B streptococci (2), group A streptococci (1), and other species and unidentified species observed by using Gram staining.

The most prevalent cause of bacterial meningitis each year was *S. pneumoniae*. No change was observed in mean number of annual cases after introduction of Hib vaccine (1997–2002, mean 74.6 annual cases vs. 2003–2009, mean 78.1 annual cases; p = 0.66). The proportion of patients co-infected with pneumococcal meningitis and HIV increased from 41.5% in 1997–2002 to 49.6% in 2003–2009 (p = 0.03), and the proportion of patients co-infected with Hib meningitis and HIV did not change after introduction of conjugate vaccine (1997–2002, 43/204 [21.1%], vs. 2003–2009, 14/56 [25%]; p = 0.53). During 2003–2009, 7 (12.5%) of 56 Hib cases were in children <14 weeks of age. We observed a decrease in the mean number of annual cases caused by *N. meningitidis* during 2003–2009 compared with 1997–2002; this organism has caused <10 cases each year since 2002.

## Conclusions

Cases of Hib meningitis in children decreased substantially in Blantyre in the postvaccination era but causes of residual disease remain unclear. We hypothesize that Hib meningitis affects mainly those not fully immunized, those with HIV, and those vaccinated before introduction of pentavalent vaccine. However, without data on vaccination status of participants, we cannot address this hypothesis and recommend more intensive research. These results complement and extend those of a previous study and provide evidence of ongoing effectiveness of the Hib conjugate vaccination program in a population with high HIV seroprevalence (*6*). Hib conjugate vaccine is not equally effective among HIV-positive patients, and the high prevalence of HIV infection may be associated with persistence of Hib meningitis (*11*). Most Hib disease is seen as pneumonia, and estimates of Hib disease incidence based on meningitis data consequently underestimate the true incidence in the general population (*2**,**12*).

Pneumococcal meningitis occurred more frequently in HIV-seropositive children than in the general population, suggesting that HIV infection is a predisposing factor. This finding has been widely reported elsewhere (*13*). Approximately 65% of children in South Africa with pneumococcal meningitis were HIV seropositive; the larger proportion of HIV co-infection in this population may be caused by the higher HIV prevalence in children in South Africa (4.5% vs. 1.7%) (*14*). Pneumococcal meningitis remains the leading cause of ABM in children in Malawi and we strongly recommend introducing the pneumococcal conjugate vaccine to reduce the incidence of ABM in this population.
